# Pharmaceutical applications of halophilic enzymes

**DOI:** 10.1016/j.heliyon.2025.e42754

**Published:** 2025-02-17

**Authors:** Maryam Yavari-Bafghi, Mohammad Ali Amoozegar

**Affiliations:** Department of Microbiology, School of Biology, College of Science, University of Tehran, Tehran, Iran

**Keywords:** Halophiles, Halophilic enzymes, Pharmaceutical applications

## Abstract

Halophiles produce extraordinarily stable enzymes under conditions where conventional enzymes cease to function, denature, and precipitate. Halophilic enzymes have adapted to function optimally at high salt concentrations besides tolerance of organic solvents and thermal stability. These special features offer potential advantages of cost-effectiveness and improved treatment outcomes that make them valuable for pharmaceutical purposes including the synthesis, design, and discovery of new drugs. The article highlights the unique characteristics and adaptation strategies of halophiles towards salinity. It provides an overview of halophilic enzymes’ pharmaceutical applications and examines different challenges, opportunities, and recent advances in applying these enzymes in this field.

## 1 Introduction

### 1.1 Halophilic enzymes; unique characteristics and potential benefits for pharmaceutical applications

Halophiles are among the most abundant extremophiles capable of growing in high-salt environments, such as salt lakes, pans, and hypersaline areas [1]. They are divided into slight, moderate, or extreme halophiles based on their salt (NaCl) requirements. The best growth occurs at 2–5% for “slight”, 5–20 % for “moderate”, and 20–30 % for “extreme” halophilic organisms [2].

In halophilic microorganisms, enzymes have evolved to function optimally under high salt conditions. Halophilic or halo enzymes have several unique features compared to their non-halophilic counterparts. Among these characteristics, salt-dependent stability, specificity, organic solvent tolerance, thermal stability, and potential for being new bioactive compounds can be mentioned [3]. Most require salt for folding and proper stability while denaturing or losing activity without salt. This salt-dependent stability makes them particularly valuable in pharmaceutical applications. Salt is vital for maintaining physiological balance in the human body (electrolyte balance, nerve function, muscle function, and blood pressure regulation) and plays a critical role in various aspects of pharmaceutical production, including stabilization, solubility enhancement, preservation, and buffering [4]. Its unique properties make it an indispensable component in both biological systems and industrial applications. Halophilic enzymes have also demonstrated more stability and activity in the presence of organic solvents. This ability of halo enzymes expands their potential application in biocatalysts and drug synthesis [3,5]. Moreover, many halophilic enzymes have remarkable thermal stability enabling them to withstand high temperatures. This thermal resistance makes them suitable for various pharmaceutical processes that require high temperatures, such as enzymatic reactions and biological transformations. For instance, chiral amine synthesis via transaminases at high temperatures is preferable as it has higher reaction output and lower production costs in the pharmaceutical industry. An estimated 40 % of pharmaceuticals have a chiral amine in their structure [6]. Additionally, the halophilic, thermo-stable protease produced by the extremely halophilic archaeon *Natrialba aegyptiaca* strain 40T and halotolerant, thermostable, and alkali-stable α-amylases from *Chromohalobacter* sp. TVSP 101 are used in pharmaceutical formulations, synthesis of peptides, and antibacterial agents [7,8]. Their ability to maintain activity and stability in high temperatures is advantageous over non-halophilic enzymes that may lose their activity under similar conditions [9]. Halophilic enzymes often show high specificity and efficiency in strong catalytic activities. Their unique adaptation to saline environments may lead to specialized active sites or structural features that increase connection and substrate recognition.

Moreover, halophilic microorganisms are exposed to unique environmental challenges that produce bioactive molecules with potential medicinal applications. These compounds include antiviral, antioxidant, osmoprotectant, antimicrobial, and anticancer agents [10]. Investigating the enzymatic properties of halophiles can generally provide therapeutic or medicinal insights. The therapeutic applications of halophilic enzymes present opportunities to synthesize, formulate, deliver, and discover new drugs. Their unique and sustainable properties in extreme conditions make them attractive candidates for various pharmaceutical applications, offering potential advantages in terms of cost-effectiveness, stability, and improved treatment outcomes [11,12]. The synthesis of chiral compounds is essential in pharmaceutical processes, often requiring costly chemical methods or regular enzymes with limited stability or characteristics. Halophilic enzymes can serve as a sustainable and cost-effective alternative for chiral compound production, providing high selectivity and performance [13]. Additionally, halophilic enzymes can be engineered or modified to exhibit the desired properties, increasing the likelihood of proper drug formation and treatment outcomes. The use of halophilic enzymes in conjunction with residues produced by pharmaceutical companies underscores their potential for bioremediation. Lastly, bioprospecting in halophilic environments may lead to the identification of new bioactive compounds with therapeutic potential [3,13].

## 2 Overview of halophilic enzymes

### 2.1 Halophilic enzymes; sources and adaptation

Halophilic enzymes are extremozymes produced by halophilic microorganisms, particularly from two major domains of life (Archaea and Bacteria) [14]. For example, the genera *Salinivibrio*, *Halomonas*, *Chromohalobacter*, *Salinicoccus*, and *Marinococcus* isolated from Salterns in Almeria, Cadiz, and Huelva (Spain), produce amylase, protease, lipase, DNase, and pullulanase [15]. In 2009, Rohban et al. [16] conducted a study in our laboratory on extremophiles in Howz Soltan, a hypersaline lake in central Iran. The organisms *Salicola, Halovibrio*, *Halomonas*, *Halobacillus*, *Gracilibacillus*, *Salinicoccus*, and *Piscibacillus* were successfully isolated and produced a wide variety of extracellular enzymes including lipase, amylase, protease, xylanase, DNase, inulinase, pectinase, cellulase, and pullulanase. In a similar study, Ghasemi et al. reported the isolation of protease and lipase obtained from *Bacillus*, *Paenibacillus*, *Halobacterium*, *Aeromonas*, and *Staphylococcus*, isolated from Maharlu Salt Lake in Iran [16,17]. In agreement with previous studies, Moreno et al. isolated the halophiles *Bacillus*, *Halobacillus*, *Pseudomonas*, *Halomonas*, and *Staphylococcus* that produce hydrolytic enzymes such as amylase, protease, lipase, DNase, xylanase, and pullulanase from the Atacama Desert (Chile) [18]. Amylase, lipase, xylanase, cellulase, and sulfate reductases are some archaeal halophilic enzymes produced from *Haloferax volcanii*, *Halomonas elongata*, *Methanohalobium evestigatum, Haloferax mediterranei, and Archaeoglobus fulgidus*, respectively [19]. In 2018, Menasria et al. [20] assigned 68 halophilic archaea to seven phylotypes within the class *Halobacteria*: *Haloarcula*, *Halococcus*, *Haloferax*, *Halogeometricum*, *Haloterrigena*, *Natrialba*, and *Natrinema*. The isolates produced amylase, cellulase, gelatinase, inulinase, lipase, esterase, pectinase, nuclease, protease, and xylanase.

These halophilic microorganisms have adapted to survive in saline environments by developing specific mechanisms to maintain osmotic balance, stabilize protein structures, and perform optimally in saline environments. Enzymes derived from these organisms often possess unique properties like increased endurance, pH and high-temperature tolerance, and resistance against denaturing agents [14]. Some of the adaptability methods of halophiles in saline environments are discussed below.

#### 2.1.1 Osmotic balance

One crucial aspect of the adaptation of halophiles in saline environments is the ability to maintain osmotic balance. Halophiles have evolved mechanisms to regulate water balance, prevent dehydration, and control their internal salt concentration to cope with high external salt levels. They accumulate high concentrations of compatible solutes, such as potassium ions, proline, and glycerol, utilize the ‘salt in’ strategy, and employ a system of active ions transport, such as sodium/proton antiporters and potassium absorption, to prevent water loss from the cells and survive in saline environments [21].

The first reported bacterial osmolyte was glycine betaine in *Halorhodospira halochloris* [22]. In high salinities, *Halobacillus halophilus* switches to producing proline as the main organic compatible solute [22]. Bacterial species like *Halanaerobium acetethylicum*, *Haloanaerobium praevalens*, and archaeal members of the families *Halobacteriaceae* and *Haloferacaceae*, such as *Halobacterium salinarum*, *Haloarcula marismortui*, *Halococcus morrhuae,* and *Haloferax* maintain their osmotic balance by concentrating K^+^ inside cells [14,[23], [24], [25], [26]]. The coordinated action of the membrane-bound proton-pump bacteriorhodopsin, ATP synthase, and the Na^+^/H^+^ antiporter generates an electrical potential that facilitates the uptake of K^+^ into cells through a K^+^ -uniport mechanism. This electrical potential must exceed the K^+^ diffusion potential for primary or secondary transporters to take up the Cl^−^ counterion [23].

#### 2.1.2 Protein and enzyme adaptation

Halophiles have developed adaptations to maintain the proteins' stability, structure, and function in high salt concentrations. They often increase the number of negatively charged amino acids (such as aspartic and glutamic acid) on the protein surface. These amino acids interact with surrounding cations and protect proteins from the destructive effects of high salt concentration. The genome sequencing of *Halobacterium* sp. NRC-1 first revealed the prevalence of negativity-charged proteomes in halophiles [24]. Similar trends, such as attaining a relatively large number of negatively charged amino acids, have been reported in protease from *Halobacterium* sp. strain LBU5030 [25], xylanase from *Planococcus* sp. SL4 [27] and α-amylases from *Haloarcula marismortui* [28].

Additionally, halophilic proteins may increase the level of hydrophobic amino acids (such as alanine and leucine) to maintain stability in high salt concentrations [29,30]. In halophilic proteins, the presence of these amino acids can help stabilize the protein structure by promoting hydrophobic interactions within the protein core. By increasing the proportion of smaller hydrophobic residues like alanine, which has a shorter side chain than larger hydrophobic residues (e.g., phenylalanine), halophilic proteins can reduce their overall hydrophobic surface area. This reduction minimizes the likelihood of aggregation and enhances solubility in saline conditions [31]. Bacteriorhodopsin a light-driven proton pump from *Halobacterium salinarum* exhibits a high degree of stability due to its unique amino acid composition, which includes an increased proportion of hydrophobic residues [31]. The presence of charged and polar residues on the surface helps maintain solubility in high-salt environments.

Halophilic enzymes also have specific variations, properties, and adaptability to improve their structure, stability, and activity in extremely saline environments. Common enzymatic adaptations observed in halophiles include increasing acidic residues on their surfaces, altering the protein and active site structure, and changing the amino acid structure [30]. For example, the halophilic β-galactosidase from *Halobacillus* features an abundance of acidic residues on its surface, facilitating interactions with salt ions and contributing to enzyme stability in high-salt environments [32,33]. Enzymes like glyceraldehyde-3-phosphate dehydrogenase from *Halobacillus trueperi*, ferredoxin from *Haloferax volcanii*, lactate dehydrogenase from *Halococcus morrhuae*, sulfate reductase from *Dehalobacter restrictus*, and malate dehydrogenase from *Haloarcula marismortui* have an increased number of acidic residues that contribute to solvation and stability, along with hydrophobic residues that help maintain their structural integrity [3,32,[34], [35], [36], [37]].

#### 2.1.3 Genome stability

Halophiles’ genomes often contain specific adaptations that enhance stability under extreme conditions. These adaptations may include genes encoding proteins and enzymes involved in DNA repair mechanisms, maintaining genomic integrity despite potential damage from environmental stressors. Repair systems include base/nucleotide excision repair, homologous recombination, and non-homologous end joining for diagnosing and repairing DNA damages such as base modifications, single- and double-strand breaks, and ensuring genome cohesion [38,39]. An interesting example is the halophilic genome sequence of *Haloarcula marismortui*, which combines features like an acidic proteome, multiple copies of general transcription factors, and multiple replicons with high G + C content [40]. The higher stability of GC base pairs in saline environments leads to higher GC content in DNA and RNA molecules of some halophiles [41]. However, the combination of these features is rare, and some may not be universal among halophiles. For example, the overall G + C content of *Halothermothrix orenii* [42], *Haloquadratum walsbyi* [43] and *Salinicoccus kunmingensis* [44] is only between 42.9 % and 47.9 %, respectively. Therefore, other specific halo adaptation characteristics need identification.

## 3 Pharmaceutical potential and application of halophilic enzymes

Halophilic enzymes are used in various fields like biotechnology, food processing, bioremediation, and pharmacology [2,45,46]. Enzymes for industrial processes must resist heat, organic solvents, and pH extremes. Halophilic organisms offer a great starting point, as they typically contain multi-stress resistant enzymes ideal for multi-extreme industrial processes. Halophilic enzymes are highly tolerant to salt, temperature, and organic solvents [3], retaining activity under extreme conditions [47], and enhancing their value for biotechnological applications [45,46]. For example, halophilic amylases from salt-loving bacteria like *Halomonas meridiana*, *Halobacillus* spp., *Micrococcus halobius*, *Halothermothrix orenii*, and *Streptomyces* sp. are used to hydrolyze starch in the food industry [8,[48], [49], [50], [51], [52]]. Halophilic proteases from *Bacillus* spp., *Pseudoalteromonas* sp., *Salinivobrio* sp., *Nesterenkonia* sp., *Halobacillus* spp., and *Chromohalobacter* sp. are applied in laundry additives, waste management, pharmaceuticals, and food processing [[53], [54], [55], [56], [57], [58]]. Most halophilic proteases are also alkaliphilic and desirable for the laundry industry [46]. Alkaliphilic, thermotolerant, and halophilic xylanases are used for paper and pulp bio-bleaching [46,59]. These enzymes were isolated from *Paraglaciecola mesophila*, *Chromohalobacter* sp., *Nesterenkonia* sp., and *Bacillus pumilus* GESF-1 [8,53,60,61]. Halophilic enzymes also show promise in bioremediation, particularly in treating residues from pharmaceutical companies and addressing environmental pollution [62]. For instance, Ng et al. tested the pharmaceutical wastewater treatment in a lab-scale anaerobic membrane bioreactor (AnMBR) and achieved COD removal of 14.7–47.2 %. The high salinity and complex nature of the organics in the wastewater caused the low organic removal efficiencies [63]. In another study, they evaluated microbial communities and demonstrated a positive influence of halophilic organisms on treatment efficiency [64]. Similarly, another report showed comparable results using mixed cultures to treat saline wastewater in an anaerobic MBR (anMBR) system. In this study, halophilic mixed cultures were used to degrade 33 trace organic compounds (trOC) belonging to the four key groups of contaminants (i.e., pharmaceuticals, personal care products, industrial chemicals, and pesticides), among them are also aromatic compounds like phenyl phenol and bisphenol A [65].

Another interesting aspect for future studies is examining and characterizing halophilic enzymes isolated from salt-loving bacteria to design enzymatic-based biosensors. Halophiles demonstrate considerable potential for heavy metal sensing in high-salinity environments and biomedical diagnostics and drug screening applications [66]. For instance, Nakayama et al. developed a whole-cell biosensor utilizing the halophilic microbe *Halomonas elongata* strain OUT30018 to detect metals in saline conditions [67]. Such novel halophilic enzymes isolated from halotolerant microorganisms could provide enzyme stability, activity, and operational capability as biosensing elements under harsh conditions, opening new opportunities for designing enzymatic biosensors [68]. Most enzyme-based biosensors developed so far have been intended for the biomedical field [69]. Extremozymes' high stability during storage and operation is advantageous for commercial applications [70]. Halophilic enzymes can also be expressed on cell surfaces using surface expression anchors, creating highly selective and sensitive microbial sensors with rapid response times.

Overall, halophilic enzymes demonstrate significant potential and compatibility in medicinal applications, thus warranting further study to explore their pharmaceutical potential and applications. Examples of specific types of these enzymes are presented below.

### 3.1 Halophilic proteases

The stability and activity in high-salt environments make halophilic proteases valuable for protein engineering and drug development [71]. The *Haloferax* species, particularly *Haloferax mediterranei* in the Archaea domain, is known for producing halophilic proteases with medicinal applications. These proteases have been studied for their ability to degrade proteins, increase drug durability, and improve the bioavailability of drug combinations [72,73]. Proteases derived from the genera *Halobacterium* such as *Hbt. salinarum* and *Hbt. halobium* has shown potential in drug delivery, wound healing, and enzymatic peptide synthesis [25]. Their capability to degrade specific biomolecules allows the drugs to be released at targeted sites, enhancing treatment efficacy. The proteolytic activity of these enzymes can assist in wound healing by promoting the removal of necrotic tissue and facilitating healthy tissue regeneration. Their incorporation into topical formulations could accelerate healing processes in chronic wounds or burns. By selectively cleaving proteins at specific sites, these enzymes can generate bioactive peptides with potential therapeutic effects, such as antimicrobial or anti-inflammatory properties [74]. The *Halococcus* species, including *Hcc. morrhuae* and *Hcc. salifodinae*, and the genus *Natrinema* like *Nnm. pallidum* and *Nnm. gari* also produces proteases potentially involved in protein modification, drug formulation, enzymatic peptide synthesis, wound healing, drug delivery, and detoxification processes [13]. The halo archaeal genus *Natrialba*, including *Nab. taiwanensis* and *Nab. magadii* also produces proteases used in pharmaceutical formulations, peptide synthesis, and antibacterial agents [7,75,76].

Proteases extracted from *Bacillus luteus* H11 have shown promise in pharmaceutical applications due to their ability to cleave peptide bonds and aid in the production and modification of bioactive peptides [77]. Additionally, extracellular protease produced by *Halobacillus andaensis* for biologically active peptide production using fish muscle as the protein source presents a great opportunity for high-value peptide production [78].

Halophilic proteases have also been used in drug discovery. These proteases can cleave peptide bonds in target proteins with high specificity and efficiency. This specificity allows for precise targeting and cleaving of particular peptide sequences within proteins of interest. Utilizing halophilic proteases to cleave target proteins, can identify key binding sites, active regions, or functional domains within the protein structure. This is crucial in investigating protein structure and function for crucial in drug target identification and validation [79,80].

Furthermore, halophilic proteases are applied in biocatalysis and bioprocessing, especially in protein modification and peptide synthesis for controlled cleavage of peptide bonds, creation of specific peptide fragments, or modification of protein structures. An extracellular protease from *Halobacterium salinarum* for peptide synthesis is a successful application of halo enzymes in a non-aqueous medium [3,5]. Halophilic proteases can also be used to treat wastewater containing residual proteins from pharmaceutical production, helping to reduce environmental pollution and improve water quality [77,81,82].

### 3.2 Halophilic lipases

In the pharmaceutical and cosmetic industries, lipases synthesize esters, fatty acid derivatives, and other lipid-based compounds. They play a crucial role in the production of lipids with specific combinations of fatty acids, which have potential applications in nutrition, drug delivery, and lipid-based formulations [13]. Additionally, these enzymes are involved in the manufacture of drug intermediates and pharmaceutically important molecules [83]. They are effective in breaking down lipid-based pharmaceutical residues, such as formulations containing oils or fats, due to their stability in saline conditions [84]. Extreme lipase is utilized in medicine and pharmaceuticals for the production of key drugs like Diltiazem hydrochloride, Epothilone A, and Nikkomycin [85]. Diltiazem hydrochloride, for instance, is commonly used for coronary vasodilation and lipase is crucial in its production using a key intermediate 3-phenylglycidic acid ester, through asymmetric hydrolysis [86].

Some *Haloarchaea* genera, including *Haloferax* and *Halorubrum*, have been studied as potential sources of halophilic lipases for medicinal applications. Archaea and bacteria such as *Haloarcula*, *Halobacterium*, *Halococcus*, and *Salinibacter* produce halophilic lipases on a large scale. Furthermore, amylase, protease, and lipase from *Halobacillus* and *Halothermothrix* have been isolated and their potential for various applications has been explored [85,[87], [88], [89], [90]].

Halophilic lipases and esterases are utilized in drug production for prodrug synthesis. The halophilic esterase isolated from *Haloarcula marismortui* and *Billgrantia gudaonensis* (formerly *Halomonas gudaonensis*) is used in the synthesis of optical compounds, perfume, and antioxidants [91]. Additionally, the halophilic lipase isolated from *Haloferax mediterranei* is also used in drug production [87].

Halophilic lipases catalyze the hydrolysis, synthesis, or modification of lipids in high salt and organic solvent concentrations. These enzymes, such as LipBL, play a crucial role in biocatalysis and bioprocessing, including producing Eicosapentaenoic Acid (EPA), biodiesel, synthesizing special lipids, and enzymatic separating of chiral compounds. LipBL, a lipolytic enzyme from the moderately halophilic bacterium *Marinobacter lipolyticus* SM19 exhibits high activity against short to medium-length acyl chain substrates and hydrolyzes olive and fish oil. LipBL enriches free eicosapentaenoic acid (EPA) to hydrolyze fish oil. Furthermore, exposure of LipBL to buffer-solvent mixtures demonstrated remarkable activity and stability in all organic solvents tested [92,93].

### 3.3 Halophilic anti-cancer/-tumor enzymes

#### 3.3.1 Halophilic asparaginase

Acute lymphoblastic leukemia (ALL) is a form of blood cancer, commonly affecting children. One crucial component in the treatment of ALL is asparaginase. Asparaginase breaks down serum asparagine into nonfunctional aspartic acid and ammonia, essentially starving tumor cells of a necessary amino acid. This interruption of asparagine-dependent protein synthesis blocks tumor cell proliferation [94]. The benefits of halophilic asparaginases, such as increased stability and reduced immunogenicity, have been explored as an alternative to conventional asparaginases [95]. Halophilic bacteria present a promising source for producing enzymes with unique immunological properties that could potentially decrease the risk of adverse immune responses associated with long-term enzyme therapy [96].

Research conducted by El-fakharany et al. [97] demonstrated the potent cytotoxic effects of L-asparaginase isolated from *Bacillus halotolerans* OHEM18, making it a promising candidate for medicinal applications as an antioxidant and antitumor drug. The cytotoxicity of L-asparaginase was tested on both normal cells (Vero cell line) and cancer cells (NFS-60, MCF-7, and HepG-2 cell lines) using the 3-(4,5-dimethylthiazol-2-yl)-2,5-diphenyltetrazolium bromide (MTT) procedure. Their findings revealed that the bacterial L-asparaginase exhibited antioxidant activity and potent antitumor efficacy. It inhibited the proliferation of various tumor cell lines by inducing the apoptosis pathway, particularly in leukemia cells [97].

In another study of our laboratory conducted by Zolfaghar et al., 110 halophilic and halotolerant bacteria were isolated from various saline environments in Iran and screened for extracellular anticancer enzyme production. Twenty-nine strains were found to produce L-asparaginase, with the highest enzyme activity observed in *Vibrio* sp. strain GBPx3 at 1.0 IU/mL [98]. Additionally, in 2017, Ghasemi et al. evaluated the anti-cancer activity of a novel recombinant L-asparaginase enzyme produced by *Halomonas elongata* strain IBRC M10216 against human lymphoblastic and myeloid leukemia cell lines, Jurkat and U937. This enzyme significantly reduced the viability of these cancer cell lines with IC50 values of 2 and 1 U/mL, respectively, while not affecting the viability of normal HUVEC cell lines [99].

#### 3.3.2 Halophilic glutaminase

Glutaminase has been considered a significant role in enzyme therapy for cancer treatment, particularly in acute lymphocytic leukemia. It has also shown effectiveness against the human immunodeficiency virus (HIV) [100]. L-glutaminase, which hydrolyzes L-glutamine to L-glutamic acid and ammonia, is used in glutamine-deprivation therapy to selectively inhibit tumor growth by blocking de novo protein synthesis and increasing superoxide levels through oxidative stress, leading to the death of cancer cells [101]. Various halophilic bacterial strains, such as *Bacillus cereus* MTCC 1305, *Bacillus subtilis*, *Providencia* sp., *Acinetobacter calcoaceticus* PJB1, *Aeromonas veronii*, and *Halomonas*, have been isolated from marine habitats for their production of L-glutaminase [98,102]. Gomaa [103] highlighted the promising features and high potential of L-glutaminase produced by the marine bacteria *Bacillus* sp. DV2-37, which has demonstrated significant antitumor activity against various carcinoma cell lines, including hepatocellular (HepG-2), human breast (MCF-7), and colon (HCT-116) cancers, indicating its potential role in cancer chemoprevention and chemotherapy [103]. In a separate study conducted in our laboratory, screening of 85 halophilic strains from the hypersaline Urmia Lake in Iran revealed that 19 % and 3.5 % of strains, primarily belonging to the genus *Bacillus* and *Salicola*, exhibited l-asparaginase and l-glutaminase activity, respectively [96]. Additionally, L-glutaminase from *Halomonas meridiana* has demonstrated significant anticancer activity against colorectal cancer cells, with potent cytotoxic effects and IC50 values of 7.0 μg/mL for LS 174T cells and 13.2 μg/mL for HCT 116 cells, indicating its effectiveness in inhibiting tumor growth and promoting apoptosis [101].

#### 3.3.3 Halophilic arginase

Halophilic arginase, an enzyme that catalyzes the hydrolysis of arginine to ornithine and urea, has significant pharmaceutical potential. By participating in the urea cycle, halophilic arginase can modulate arginine metabolism and production, offering potential therapeutic benefits in conditions associated with urea cycle disorders or arginine imbalances [104]. In a study conducted in our laboratory, Zolfaghar et al. [98] screened for halophilic and halotolerant anti-cancer l-arginase-producing bacteria, considering the lower immunological reactions of halophilic or halotolerant enzymes in patients. Two out of 110 halophilic and halotolerant strains produced l-arginase. The maximum enzyme activity was observed in strain GAAy3 belonging to the genus *Planococcus*, with l-arginase activity at 3.1 IU/ml. In another study, Unissa et al. demonstrated purified l-arginase from the halophilic bacterial species *Idiomarina sediminum*; H1695 showed good activity against HeLa cells with an IC50 value of 0.5U/ml. Therefore, l-arginase can be a potential candidate as an anticancer agent [105].

#### 3.3.4 Halophilic transferase

In recent years, nucleoside phosphorylases have gained increasing attention for their biocatalytic potential in synthesizing antiviral and anticancer drugs, such as Islatravir [106]. A significant challenge in these enzymatic reactions is the poor water solubility of the substrates, including nucleobases and sugar donors. This limitation often requires high co-solvent concentrations, which can lead to enzyme inactivation. Recent studies have highlighted *Halomonas elongata* as a valuable source of robust enzymes for overcoming these challenges. Two specific enzymes, a purine nucleoside phosphorylase (HePNP) and a thymidine phosphorylase (HeTP), have been characterized for their impressive stability in organic solvents. HePNP retains about 50 % activity after three days in 50 % dimethyl sulfoxide (DMSO), while HeTP maintains 80 % activity after 3 h in 10 % ethanol [107]. The operational stability of both enzymes upon immobilization on porous particles has enabled the synthesis of valuable pharmaceutical molecules, achieving the highest reported yields for these enzymatic reactions [108].

### 3.4 Halophilic glycosyltransferases

Halophilic glycosyltransferases are used in drug discovery to synthesize glycosylated compounds. These enzymes can create glycosylated derivatives of drugs by adding sugar to specific parts of drug molecules. In general, one of their important roles is in the biosynthesis of glycosidic linkages by transferring sugar residues from donor substrates to acceptor molecules. These acceptor substrates include mono-, di-, or oligosaccharides, proteins, lipids, DNA, and various small molecules. As a result, these enzymes play pivotal roles in the biosynthesis pathways of oligosaccharides and polysaccharides, protein glycosylation, and the formation of valuable natural products. The mechanism by which glycosyltransferases achieve regio- and stereo-specific transfer of sugars can occur through an inverting or retaining mechanism, determining the stereochemical outcome (α- or β-glycosides) [109]. Therefore, glycosyltransferases facilitate the site-specific conjugation of glycan residues to therapeutic molecules, enhancing their delivery to specific tissues or cells. By attaching glycan moieties to drugs or imaging agents, glycosyltransferases enable the development of bioconjugates that can serve dual purposes—therapeutic action and diagnostic imaging [110]. Glycosylation can significantly improve the solubility, stability, and bioavailability of drugs, and it plays a crucial role in the glycosylation of natural products, leading to the discovery of new drugs with enhanced biological activities [111]. *Haloferax mediterranei* produces a halophilic glycosyltransferase (CGTase) that facilitates the synthesis of glycosylated compounds in saline environments [112].

### 3.5 Halophilic uricase

Uricase or urate oxidase is a therapeutic enzyme that breaks down uric acid into the more soluble compound allantoin. This process helps excrete excess uric acid from the body and treat related illnesses. The enzyme oxidatively changes uric acid to allantoin, CO_2_ (Carbon dioxide), and H_2_O by unlocking its purine ring [113,114]. Allantoin, a soluble molecule in plasma, can be easily discarded through the kidneys. It is 5–10 times more soluble than uric acid but is not naturally present in humans [115].

One method for producing uricase is through PEGylation, where purification is performed using three sequential chromatographic columns. Rasburicase (Fasturtec®) is a recombinant form of uricase of *Aspergillus flavus* origin. It is primarily used for preventing and treating intense hyperuricemia resulting from acute lymphoblastic leukemia in children [116] and is highly effective in treating tumor lysis syndrome. As one of the first attempts, Aly et al. separated *Streptomyces exofolitus* from the soil, which was found to be a high producer of uricase [117]. In 2024, Nasir Shirazi et al. investigated potential sources of uricase production from the Eshtehard salt desert in the Alborz province of Iran. In our laboratory, they conducted heterologous expression, purification, and functional assays of the enzyme, demonstrating the efficient production and purification of recombinant uricase from an enzyme-producing *Streptomyces* strain in *E. coli* [118]. In a similar study, our team screened fifty-eight halophilic strains isolated from Iranian saline lakes for uricase production. Eighteen strains showed positive uric-lytic activity, while *Halobacillus* sp. strain GCFx14 exhibited the highest uricase activity at 0.131 U/ml [119].

### 3.6 Halophilic streptokinase

Streptokinase is an enzyme widely used to dissolve blood clots in treating thrombosis. These clots, made of insoluble fibrin, restrict blood flow in blood vessels and can lead to thrombosis and heart attacks, resulting in a high number of deaths each year. Currently, three fibrinolytic (thrombolytic) agents are used worldwide: Streptokinase, urokinase, and tissue plasminogen activator (t-PA) or plasminogen activator (PA).

Streptokinase forms a complex with plasminogen, converting it into plasmin. This conversion is crucial because plasmin is the active enzyme responsible for breaking down fibrin clots, the main components of blood thrombi [120]. However, these enzymes can cause adverse side effects such as gastrointestinal bleeding and allergic reactions, in addition to being expensive, heat sensitive, having low stability, and requiring high therapeutic doses [121]. Halophilic streptokinase, with unique features and reduced immunogenicity, is suitable for medicinal applications, particularly in thrombosis treatment. Furthermore, it has been reported that this enzyme has various other applications in clinical, industrial, and food sectors such as blood pressure regulation, proteolysis, and anti-inflammatory properties [122]. Halophilic *Streptomyces flaveolus* and *Streptomyces galtieri* produce fibrinolytic enzymes that have the potential to be used as thrombolytic agents [121].

### 3.7 Halophilic hyaluronidase

Hyaluronidase is an enzyme that breaks down hyaluronic acid (hyaluronan), the main component of the extracellular matrix and connective tissue. This enzyme functions as an endoglycosidase that cleaves the glycosidic bonds in hyaluronic acid, breaking it into smaller fragments [123]. It has long been successfully used in cosmetic medicine and wound healing to increase the absorption of drugs into tissue and reduce tissue damage in cases of drug extravasation. With the increasing popularity of hyaluronic acid filler, hyaluronidase has become essential for correcting complications and unsatisfactory results after filler injection [123,124]. In a study by Han et al. [125], a halophilic hyaluronidase was isolated from marine bacteria and expressed recombinantly. This enzyme was stable at 0 to 40 °C for up to 24 h and its activity was independent of divalent metal ions.

### 3.8 Halophilic amylases

Halophilic amylases hydrolyze starch and glycogen into simpler sugars. These enzymes have various pharmaceutical applications including producing glucose syrups used as excipients in pharmaceutical formulations, modifying starches for controlled drug release systems, and synthesizing oligosaccharides with potential therapeutic properties [126,127]. One of the main applications of α-amylases is starch saccharification as an effective biocatalyst to produce maltooligosaccharides (MOS) with 3–10 glucose molecules linked by α-1,4 glycosidic bonds. MOS is commonly used in the food and pharmaceutical industries [128]. Various species of *Haloarcula*, *Halobacterium*, *Halothermothrix*, *Micrococcus*, *Natronococcus*, *Halomonas*, *Nesterenkonia*, and *Haloferax* can produce halophilic amylases [8,129,130]. Additionally, these enzymes sourced from *Haloarcula* species have shown potential for use in pharmaceutical processes that involve high temperatures and saline conditions, further expanding their applicability in the industry [129].

Furthermore, in the pharmaceutical industry, halophilic cyclodextrin glucosyltransferases are used to improve the lifetime of different drugs and decrease the effective dose. These proteins generate cyclodextrins, which can form inclusion bodies to eliminate toxic compounds [131]. Additionally, halophilic amylases can degrade pharmaceutical residues containing starch-based excipients or other polysaccharides, facilitating the breakdown of complex organic pollutants in wastewater treatment processes. Their activity in high-salt environments makes them suitable for bioremediation in saline conditions [47]. α-Amylase isolated from a moderately halophilic *Marinobacter* sp. EMB8 showed significant activity in high-salt environments. The immobilized α-amylase retains high activity for starch hydrolysis, making it suitable for degrading related pharmaceutical residues in saline wastewater [132]. *Chromohalobacter* sp. TVSP 101 produces two forms of amylases whose secretion is salt-dependent, alkali-stable, and moderately thermophilic. These amylases may have applications in hydrolyzing starch under high-stress conditions in juices or syrups that have high concentrations of sucrose or other solutes, and they may be useful for treating saline water or waste solutions containing starch residues in the presence of high salt [8].

Extracellular halophilic, alkali, and thermostable gluco-amylo-pullulanase from *Amphibacillus* sp. NM-Ra2 is resistant to surfactants, oxidizing agents, and organic solvents. This enzyme can be utilized in starch processing, pharmaceuticals, food, and paper/pulp industries. Additionally, alkaline amylases are essential for producing cyclodextrins in the pharmaceutical industry [127].

### 3.9 Halophilic oxidoreductases

Halophilic oxidoreductases, including laccases, hydrogenases, alcohol dehydrogenase (ADH2), glucose and glutamate dehydrogenases (GDHs), are utilized in drug production for pharmaceutical intermediates. These enzymes are crucial for oxidizing substrates and reducing electron acceptors, which is vital for energy production in halophilic organisms. They also aid in the addition or removal of specific functional groups in drug molecules [133,134]. Examples of highly salt/solvent resistant laccases include those from the extremely halophilic archaeon *Haloferax volcanii* and the moderately halophilic bacterium *Aquisalibacillus elongatus*, along with glutamate dehydrogenase from *Halobacterium salinarum*, all of which have been employed in the pharmaceutical industry [133]. In addition, halophilic alcohol dehydrogenase (ADH2) from *Haloferax volcanii* retains 47 % activity in 30 % DMSO and 38 % in 30 % MeOH after 72 h, making it appealing to industry due to its organic solvent tolerance [134]. HeADH-II from *Halomonas elongata*, in conjunction with NADH-oxidase from *Lactiplantibacillus pentosus* (formerly *Lactobacillus pentosus*) (LpNOX), has shown 92 % and 82 % activity in 10 % and 20 % (*v*/*v*) DMSO, respectively [135]. Glucose dehydrogenases are also widely used in the pharmaceutical industry to produce medicines [136]. Moreover, the potential use of several amino acid dehydrogenases in diagnostic kits and biosensors and chiral amino acid synthesis for the pharmaceutical industry has been reported. Halophilic amino acid dehydrogenases for the mentioned applications could provide major benefits of shelf-life stability and durability [3]. Rinaldi et al. [137] presented a novel experimental approach to produce high-quality Glutamate dehydrogenase monolayers for nano biosensing applications; their finding powerfully suggests one-step soft lithography as a powerful technique to realize both micropatterned and physiologically self-assembled layers with biocompatible properties for cell culture applications in both basic biology studies and biosensors, drug discovery, and hybrid devices.

Halophilic reductases, particularly those involved in critical metabolic processes such as anaerobic respiration, cause the reduction of nitrogenous compounds (nitrate, nitrite), oxy chlorates, ammonium, DMSO/sulfoxides, iron, and heavy metals, play a vital role in bioremediation efforts targeting pharmaceutical residues and other contaminants [133,138]. For instance, nitrogenous compounds and chlorate reductase activity of *Haloferax mediterranei* can be harnessed for bioremediation processes aimed at detoxifying nitrogenous/chlorate-contaminated wastewater, including pharmaceutical residues that may contain nitrogenated/chlorinated compounds [139,140]. *Haloferax mediterranei* is a denitrifying haloarchaeon using nitrate as a respiratory electron acceptor under anaerobic conditions in a reaction catalyzed by pNarGH. Other ions such as bromate, perchlorate, and chlorate can also be reduced [139,140]. In the Nájera-Fernández et al. study [141], this archaeon can remove 60 % nitrate and 75 % of the nitrite initially present in brine samples collected from a wastewater treatment facility. These results suggest that *H. mediterranei*, and probably other halophilic denitrifying Archaea, are suitable candidates for the bioremediation of brines with high nitrite and nitrate concentrations. In a similar study by our team, a halophilic strain identified as *Kocuria rosea R3A34* was isolated from saline environments in Iran. This strain expresses the narG and napA genes, which encode for membrane-bound and periplasmic nitrate reductases, respectively. The strain demonstrated high nitrate-reducing capacity, making it a promising candidate for treating saline wastewater containing nitrate pollutants from agricultural and pharmaceutical sources [142]. In previous studies, nitrate reductase's ability to function in high-salinity conditions was also reported from *Haloferax alexandrines* and *Haloarcula marismortui* [143,144]. In addition, halophilic ammonium-oxidizing bacteria such as *Nitrosomonas marina*, *Nitrosomonas europaea,* and *Nitrosococcus mobilis* have been isolated from saline environments. These bacteria oxidize ammonium to nitrite, playing a crucial role in the nitrogen cycle [145]. *Bacillus tequilensis* and strains such as *Alcaligenes* have been identified as halophilic heterotrophic nitrifying bacteria capable of removing ammonium. They can utilize organic carbon sources while oxidizing ammonium to nitrite and nitrate. They can be applied in bioremediation strategies to remove ammonium from wastewater, including effluents containing pharmaceutical residues [146,147].

The DMSO reductase superfamily, found in various halophilic and extremophilic organisms, catalyzes redox reactions involving DMSO and other sulfur compounds. Their broad substrate specificity allows them to be utilized in bioremediation strategies to target pharmaceutical residues containing DMSO and related compounds [148]. The DMSO reductases from the *Rhodobacter* genus, *Haloferax volcanii*, and *Haloarchaea* can be utilized in bioremediation processes to convert harmful DMSO into less toxic compounds, thus aiding in treating pharmaceutical waste [[149], [150], [151], [152], [153]]. Furthermore, the ability of *Halomonas venusta* [154], *Bacillus pumilus* [155], *Halobacterium salinarum* [156], *Haloferax mediterranei* [157,158], and *Halomonas elongata* [159] to detoxify heavy metals such as lead (Pb) and cadmium (Cd), iron (Fe), copper (Cu), barium (Ba), and mercury (Hg) makes them a suitable candidate for the bioremediation of wastewater containing pharmaceutical residues with high concentrations of these metals.

### 3.10 Halophilic esterase/transaminases

These halophilic enzymes hydrolyze compounds containing ester, amide, and thioester bonds and transfer amino groups between molecules. They activate or detoxify prodrugs, convert substrates to desired products, and synthesize chiral amines [3,160]. A few esterases from *Halomonas elongata* have potential applications in the pharmaceutical and biomedicine industry. One of these esterases, HeE, has been used to hydrolyze non-steroidal anti-inflammatory drugs (NSAIDs) in the presence of a 10 % organic co-solvent [161]. Another example is a new esterase belonging to the YbfF family that presented a maximum activity at high salt exposure such as 0.5–4 M NaCl [162]. Halophilic transaminases are used in synthesizing pharmaceuticals and fine chemicals, particularly chiral amines such as α-, *ß*-, and ω-amino acids, synthetic amino acids, and primary amines. An estimated 40 % of pharmaceuticals contain a chiral amine in their structure [6,163]. An alternative to traditional synthetic approaches for producing betazole drugs involves a one-pot biocatalytic system that uses an ω-transaminase, an alcohol dehydrogenase, and a water-forming NADH oxidase for in situ cofactor recycling. This system achieves a 75 % molar conversion in batch reactions with soluble enzymes.

HewT, the transaminase from the moderately halophilic bacterium *Halomonas elongata* DSM 2581, is highly (S)-selective and capable of completely converting (S)-1-phenylethylamine to acetophenone, with no activity on the corresponding (R)-1-phenylethylamine. This enzyme can tolerate up to 20 % cosolvents and is a promising candidate for pharmaceutical and industrial applications [163]. HewT has unique properties that other homologous transaminases lack, making it valuable for synthesizing high-value molecules in pharmaceutical synthesis [[164], [165], [166], [167], [168]]. Table 1 provides examples of specific types of halophilic enzymes with pharmaceutical applications.

**Table 1:** Examples of specific types of halophilic enzymes with pharmaceutical applications.

NO.	Halophilic Enzymes	Applications	Microorganisms	References
1	Proteases	Drug durability increases and the bioavailability of drug combinations improve	*Haloferax mediterranei*	[72,73]
		Drug delivery, wound healing, enzymatic peptide synthesis^a^	*Halobacterium*	[25]
		Protein modification, drug formulation, enzymatic synthesis of peptides, wound healing, drug delivery, and detoxification processes^a^	*Halococcus, Natrinema*	[13]
		Pharmaceutical formulations, synthesis of peptides, and antibacterial agents	*Natrialba*	[7,75,76]
		Bioactive peptide production and modification	*Bacillus luteus* H11	[77]
		Peptide production	*Halobacillus andaensis*	[78]
2	Lipases	Synthesis of optical compounds, perfume, and antioxidants	*Haloarcula marismortui, Billgrantia gudaonensis*	[91]
		Drug production	*Haloferax mediterranei*	[87]
		Biocatalysis and bioprocessing	*Marinobacter lipolyticus* SM19	[92,93]
3	Asparaginase	Antioxidant and antitumor drug	*Bacillus halotolerans* OHEM18	[97]
		Anticancer agent	*Halomonas elongata*	[99]
		Anticancer enzyme	*Vibrio* sp. strain GBPx3	[98]
		Anti-cancer activity	*Halomonas elongata* strain IBRC M10216	[99]
4	Glutaminase	Cancer chemoprevention and chemotherapy	*Bacillus* sp. DV2-37	[103]
		Cancer treatment	*Bacillus cereus* MTCC 1305, *Bacillus subtilis*, *Providencia* sp., *Acinetobacter calcoaceticus* PJB1, *Aeromonas veronii*, *Salicola*, *Halomonas*	[98,102]
		Anticancer activity against colorectal cancer cells	*Halomonas meridiana*	[101]
5	Arginase	Anti-cancer agent	*Planococcus* GAAy3	[98]
			*Idiomarina sediminum; H1695*	[105]
6	Uricase	Intense hyperuricemia treatment	*Aspergillus flavus*	[116]
		Tumor lysis syndrome treatment	*Streptomyces exofolitus*	[117]
		Uric-lytic activity	*Halobacillus* sp. strain GCFx14	[119]
7	Streptokinase	Thrombolytic agents^a^	*Streptomyces flaveolus*, *Streptomyces galtieri*	[121]
8	Amylases	Maltooligosaccharides (MOS) production with therapeutic properties^a^	*Haloarcula*, *Halobacterium*, *Halothermothrix*, *Haloferax, Micrococcus, Natronococcus, Halomonas, Nesterenkonia*	[8,129,130]
		Bioremediation efforts targeting pharmaceutical residues	*Marinobacter* sp. EMB8 *Chromohalobacter* sp. TVSP 101 *Amphibacillus* sp. NM-Ra2	[8,127,132]
9	Oxidoreductases	Drug production for pharmaceutical intermediates	*Haloferax volcanii*, *Aquisalibacillus elongatus, Halobacterium salinarum*	[133]
		Bioremediation efforts targeting pharmaceutical residues	*Haloferax mediterranei* *Kocuria rosea R3A34* *Haloferax alexandrines* *Haloarcula marismortui* *Nitrosomonas marina* *Nitrosomonas europaea* *Nitrosococcus mobilis* *Bacillus tequilensis* *Alcaligenes* *Rhodobacter* *Haloferax volcanii* *Halomonas venusta* *Bacillus pumilus* *Halobacterium salinarum* *Halomonas elongata*	[139,140] [142] [143,144] [145] [146,147] [[149], [150], [151], [152], [153]] [154] [155] [156] [157,158] [159]
10	Glycosyltransferases	Drug's efficacy, stability, and bioavailability	*Haloferax mediterranei*	[112]
11	Esterase	Hydrolysis of non-steroidal anti-inflammatory drugs (NSAIDs)	*Halomonas elongata*	[161]
12	Transaminases	Synthesizing pharmaceuticals and fine chemicals	*Halomonas elongata DSM 2581*	[163]

a Only suggested.

Considering this, halophilic enzymes are valuable biocatalysts prepared from microorganisms adapted to saline environments. The above examples present the diverse applications of halophilic enzymes in drug synthesis, discovery, and bioprocessing. Their unique properties, such as stability in high-salt conditions, tolerance of organic solvents, and adaptability to different situations, make them attractive for various enzymatic processes in biotechnology and pharmaceutical applications.

## 4 Challenges and opportunities

### 4.1 Genetic engineering and directed evolution of halophilic enzymes

Genetic engineering and halophilic enzyme's directed evolution come with challenges and opportunities. These enzymes often have unique structural features and can adapt to high-salt concentration environments. Nevertheless, their complexity can make it difficult to understand structure-function relationships, which complicates predicting how genetic changes affect them. On the one hand, maintaining enzyme activity and stability in suboptimal saline conditions or the absence of salt can be challenging for their practical applications. Conversely, genetic engineering and directed evolution provide opportunities to increase the performance of halophilic enzymes. Through targeted mutations or directed evolution approaches, enzyme properties such as activity, stability, substrate specificity, or other new functions can be improved to meet specific application needs. Genetic engineering and directed evolution also open opportunities to produce halophilic enzymes with new activities and increase their performance in industrial environments. Optimizing these enzymes through genetic engineering and directed evolution can help to develop environmentally friendly and efficient biotechnological processes [169].

### 4.2 Protein stability and salt tolerance

Various challenges such as maintaining stability and catalytic activity in non-optimal salt conditions and preventing protein aggregation are relevant for the practical applications of halophilic enzymes. However, by understanding the factors affecting enzyme stability and activity in suboptimal salt conditions and with the help of protein engineering techniques, it is possible to expand the practical applications of halophilic enzymes in different conditions [170,171].

### 4.3 Scale-up and industrial production

Choosing suitable expressive systems and hosts for the large-scale production of halophilic enzymes can be challenging. Halophilic enzymes may have specific requirements, such as high salt concentration, extreme pH, or other conditions that must be considered when selecting expressive systems. Identifying compatible hosts that can efficiently produce and secrete enzymes is crucial. Another significant challenge is maintaining affordability while increasing the production of halophilic enzymes. Limitations associated with the overexpression of halophilic enzymes in bacterial hosts, such as *Escherichia coli*, can significantly affect the feasibility and efficiency of industrial production processes. When overexpressed in *E. coli*, halophilic enzymes often accumulate as inclusion bodies, which are insoluble aggregates of misfolded proteins. This phenomenon occurs because the folding and post-translational modifications required for proper enzyme activity may not efficiently occur in the *E. coli* cytoplasm, particularly for enzymes that require high salt concentrations for stability and activity. Inclusion bodies must be solubilized and refolded to recover active enzymes, which adds complexity and cost to the production process. In other words, refolding solubilized inclusion bodies into their functional forms can be challenging. The conditions required for proper folding (e.g., ionic strength, pH, and temperature) may differ significantly from those used during bacterial growth [172,173]. Moreover, achieving the correct three-dimensional structure is critical for enzymatic activity, and improper folding can lead to loss of function or reduced catalytic efficiency. In addition, halophilic enzymes are adapted to extreme conditions that may not be compatible with standard laboratory expression systems. For instance, the optimal conditions for enzyme activity (high salt concentrations) can inhibit the growth of *E. coli*, making it difficult to achieve high cell densities necessary for large-scale production. This incompatibility can limit the yield and viability of halophilic enzymes expressed in non-native hosts [173]. To address these challenges, several strategies can be implemented including adjusting growth conditions (e.g., using high-salt media), utilizing alternative hosts (halophilic archaea or other extremophiles), and employing genetic engineering techniques to enhance the solubility and activity of halophilic enzymes during expression, improve yield and functionality, and increase expression levels and functional properties, respectively [174]. The production process must be optimized to maximize enzyme yield, minimize production costs, and ensure quality. Strategies should also maintain enzyme stability and activity during large-scale production, purification, and storage to preserve optimal performance. By optimizing the production process and downstream processing techniques, reducing costs, and enhancing protein stability, it is possible to increase efficiency, improve quality, and produce cost-effective and durable enzymes [25].

## 5 Recent advances and future directions

### 5.1 Metagenomic approaches for enzyme discovery

In the last decade, Next-Generation Sequencing (NGS) has been the most significant event in microbial ecology and biotechnology. The advancement of this technology has led to the creation of a new field called metagenomics. While genomics involves analyzing the genomic DNA of a single organism or cell, metagenomics refers to surveying genomic DNA obtained from an entire population. Metagenomics methods provide the possibility of examining the composition of a microbial community with high efficiency and without the need for time-consuming cultivation and purification steps. Metagenomics provides more extensive data on the genomic relationship between function and organism phylogeny by accessing the functional genes of microbial communities. This is compared to phylogenetic studies, which often focus only on the diversity of conserved genes. Uncultivated organisms provide genetic information about new catalysts or enzymes [175,176]. Various metagenomics approaches for enzyme discovery take advantage of the enormous genetic diversity of microbial communities and enable the identification of novel enzymes with potential applications in different fields, including biotechnology, pharmaceuticals, biofuels, and environmental remediation. Some of the approaches for enzyme discovery using metagenomics are as follows.

• Construct a metagenomic library by directly extracting and cloning DNA from environmental samples
• Sequence-based and functional screening of desired enzyme activities
• Bioinformatic analysis
• Metagenomics data mining
• Functional expression and characteristics
• Metagenomic enzyme engineering.

These approaches have significantly accelerated the discovery and development of enzymes with unique functions and improved properties [177,178].

### 5.2 Bioinformatics and computational tools for enzyme prediction

Bioinformatics and computational tools play an important role in enzyme prediction and identification. These tools utilize different algorithms, databases, and analysis methods to identify potential enzyme-encoding genes, predict their function, and gain insight into their properties [179]. Some bioinformatics methods and common computing tools for predicting enzymes are mentioned below.

#### 5.2.1 Sequence annotation tools

BLAST (Basic Local Alignment Search Tool) and HMMER (Hidden Markov Model-based sequence similarity search) compare sequences to databases of known enzymes or protein families. These tools give information on sequence similarity, functional domains, and conserved motifs aiding in enzyme annotation and classification [180,181].

#### 5.2.2 Protein structure prediction

Homology and ab initio modeling, along with threading algorithms are applied to predict the 3D structure of enzymes, aiding in understanding catalytic mechanisms, substrate binding sites, and key residues involved in enzymatic activity [182].

#### 5.2.3 Functional annotation databases

UniProt, Pfam, and InterPro Databases provide information on protein families, domains, and functional annotations, which can be queried to obtain enzyme functional annotations for biochemical activities and substrate prediction [183].

#### 5.2.4 Metabolic pathway analysis

KEGG (Kyoto Encyclopedia of Genes and Genomes) and MetaCyc supply information on metabolic pathways and enzyme-catalyzed reactions. They can infer potential enzymatic functions and predict the role of enzymes in metabolic networks through knowledge of the genomic context and pathway information [184,185].

### 5.3 Synthetic biology applications in halophile research

Synthetic biology can engineer and reprogram biological systems and, therefore, has many applications in the field of halophilic enzyme research, including.

#### 5.3.1 Genetic engineering of halophiles

Synthetic biology can engineer salt tolerance mechanisms, modify metabolic pathways, introduce genetic circuits, and optimize gene expression, to improve the halophiles and their enzyme performance in various fields and optimization of the halophilic enzyme production [186].

#### 5.3.2 Biosynthesis of compatible solutes

Compatible solutes are organic compounds produced by halophiles to deal with the harmful effects of high salt concentration. Synthetic biology can be used to engineer heterologous pathways for compatible solute biosynthesis in non-halophilic organisms [187].

#### 5.3.3 Metabolic engineering for biological products

Synthetic biology enables the engineering of metabolic pathways in halophiles to produce valuable compounds such as biofuels, bioplastics, and pharmaceutical products [188].

## 6 Conclusion

Pharmaceutical applications of halophilic enzymes represent an exciting frontier in biotechnology and drug discovery. Halophiles that produce extraordinarily stable enzymes under harsh conditions provide a unique source of biocatalysts with several advantages over traditional enzymes. The halophilic enzymes' adaptability to high salt concentrations, organic solvents, and thermal stresses demonstrates their robustness and provides new avenues for cost-effective and efficient pharmaceutical applications.

The abundance of halophilic enzymes offers a valuable resource for drug synthesis, design, and discovery, creating an opportunity for further innovation to enhance treatment outcomes in the pharmaceutical industry. This review focuses on the unique characteristics and adaptation strategies of halophiles to salinity, highlighting the crucial role of their enzymes in pharmaceutical research and development. It also explores the challenges, opportunities, and recent advancements related to the potential of halophilic enzymes in drug design, biocatalysis, and therapeutic interventions. Continued research and utilization of halophilic enzymes could lead to improved healthcare, treatment efficiency, and the advancement of pharmaceutical science, shaping the industry's future.

## CRediT authorship contribution statement

**Maryam Yavari-Bafghi:** Writing – review & editing, Writing – original draft. **Mohammad Ali Amoozegar:** Writing – review & editing, Validation, Conceptualization.

## Declaration of competing interest

The authors declare that they have no known competing financial interests or personal relationships that could have appeared to influence the work reported in this paper.
